# Prediabetes Conversion to Normoglycemia Is Superior Adding a Low-Carbohydrate and Energy Deficit Formula Diet to Lifestyle Intervention—A 12-Month Subanalysis of the ACOORH Trial

**DOI:** 10.3390/nu12072022

**Published:** 2020-07-07

**Authors:** Martin Röhling, Kerstin Kempf, Winfried Banzer, Aloys Berg, Klaus-Michael Braumann, Susanne Tan, Martin Halle, David McCarthy, Michel Pinget, Hans-Georg Predel, Jürgen Scholze, Hermann Toplak, Stephan Martin, ACOORH Study Group

**Affiliations:** 1 West-German Center of Diabetes and Health, Düsseldorf Catholic Hospital Group, 40591 Düsseldorf, Germany; kerstin.kempf@wdgz.de (K.K.); stephan.martin@vkkd-kliniken.de (S.M.); 2 Department of Sports Medicine, Institute for Sports and Sport Science, University of Frankfurt, 60487 Frankfurt, Germany; banzer@sport.uni-frankfurt.de; 3 Faculty of Medicine, University of Freiburg, 79117 Freiburg, Germany; berg.aloys@web.de; 4 Department of Sports and Movement Medicine, Faculty of Psychology and Human Movement Sciences, University of Hamburg, 20148 Hamburg, Germany; braumann@uni-hamburg.de; 5 Department of Endocrinology, Diabetes and Metabolism and Division of Laboratory Research, University Hospital Essen, University Duisburg-Essen, 45122 Essen, Germany; susanne.tan@uk-essen.de; 6 Department of Prevention, Rehabilitation and Sports Medicine, Klinikum rechts der Isar, Technical University of Munich (TUM), 80992 Munich, Germany; Martin.Halle@mri.tum.de; 7 DZHK (German Centre for Cardiovascular Research), Partner Site Munich Heart Alliance, Munich, Germany; 8 Public Health Nutrition Research Group, London Metropolitan University, London N7 8DB, UK; d.mccarthy@londonmet.ac.uk; 9 Department Endocrinologie, Diabete et Maladies Métaboliques, Faculte de Medicine de‘l University de Strasbourg, 67170 Strasbourg, France; c.seyller@ceed-diabete.org; 10 Institute of Cardiovascular Research and Sports Medicine, German Sport University Cologne, 50933 Cologne, Germany; predel@dshs-koeln.de; 11 KARDIOS, Cardioligists in Berlin, 10787 Berlin, Germany; juergen.scholze@yahoo.de; 12 Department of Medicine, Division of Endocrinology, Medical University of Graz, 8010 Graz, Austria; hermann.toplak@medunigraz.at; 13 Faculty of Medicine, Heinrich Heine University Düsseldorf, 40591 Düsseldorf, Germany

**Keywords:** prediabetes, lifestyle intervention, formula diet, low-carbohydrate, multicenter study, RCT

## Abstract

Lifestyle interventions have been shown to reverse hyperglycemia to normoglycemia. However, these effects are not long-lasting and are accompanied with high dropout rates. As formula diets have been shown to be simple in usage and effective in improving glycemic control, we hypothesised that adding a low-carbohydrate and energy deficit formula diet to a low-intensity lifestyle intervention is superior in reversing prediabetes compared with lifestyle intervention alone. In this predefined subanalysis of an international, multicenter randomised controlled trial (*Almased Concept against Overweight and Obesity and Related Health Risk* (ACOORH) study (ID DRKS00006811)), 141 persons with prediabetes were randomised (1:2) into either a control group with lifestyle intervention only (CON, *n* = 45) or a lifestyle intervention group accompanied with a formula diet (INT, *n* = 96). Both groups were equipped with telemonitoring devices. INT received a low-carbohydrate formula diet substituting three meals/day (~1200 kcal/day) within the first week, two meals/day during week 2–4, and one meal/day during week 5–26 (1300–1500 kcal/day). Follow-up was performed after 52 weeks and 105 participants (75%, INT: *n* = 74; CON: *n* = 31) finished the 26-week intervention phase. Follow-up data after 52 weeks were available from 93 participants (66%, INT: *n* = 65; CON: *n* = 28). Compared with CON, significantly more INT participants converted to normoglycemia after 52 weeks (50% vs. 31%; *p* < 0.05). The risk reduction led to a number-needed-to-treat of 5.3 for INT. Lifestyle intervention with a low-carbohydrate formula diet reduces prediabetes prevalence stronger than lifestyle intervention alone and is effective for type 2 diabetes prevention.

## 1. Introduction

Energy deficit diets in liquid form have been shown to be an effective and feasible approach to treat obesity associated type 2 diabetes [1,2,3,4], improving cardiometabolic risk factors such as waist circumference, fat mass, blood pressure, insulin, or HbA1c [5,6]. Moreover, intervention studies with very low-energy formula diets have been shown to induce diabetes remission [1,7,8,9,10]. As a consequence, formula diets have been included in current guidelines for baseline treatment of type 2 diabetes [11,12,13]. These guidelines recommend food substitution by low-energy (≈ 800–900 kcal per day) formula diets followed by gradual reintroduction of food accompanied with intensive, sustained counseling.

Moreover, several studies have shown that a reduced carbohydrate intake [14] or a reduced number of carbohydrate-containing meals lead to improvements in glucose metabolism [15]. Furthermore, a recent intervention study demonstrated that a low-carbohydrate diet in participants with overweight and obesity can lead to an increased energy expenditure compared with a high-carbohydrate diet [16]. In contrast, there is emerging evidence that especially carbohydrate-rich nutrition inducing hyperinsulinemia is a key driver in the progression of normoglycemia to hyperglycemia [16,17,18,19]. Mechanistic studies have explained this phenomenon by demonstrating that consuming high amounts of carbohydrates causes impairments in glucose metabolism and associated organs [18,20].

However, there is still controversy whether a low-carbohydrate and moderate-energy meal replacement therapy by formula diet can convert prediabetes into normoglycemia, as this has only been shown before in uncontrolled [21] or small sample size intervention trials [5,22]. Therefore, a multicenter randomised controlled trial, the *Almased Concept against Overweight and Obesity and Related Health Risk* (ACOORH) study, was initiated to investigate the additional effect of a low-carbohydrate formula diet on top of a low-intensity lifestyle intervention in comparison with a lifestyle intervention alone in a larger cohort of high-risk individuals with obesity. In the present study, we report on the predefined subanalysis of the ACOORH study, focusing on the effect of a low-carbohydrate and energy deficit formula diet in combination with a low-intensity lifestyle intervention compared with lifestyle intervention alone on the conversion rate to normoglycemia in patients with prediabetes.

## 2. Materials and Methods

### 2.1. Study Design

In this predefined subanalysis of the ACOORH trial, individuals with prediabetes and overweight or obesity (defined as HbA1c: 5.7–6.49% [39–46 mmol/mol]) (*n* = 141, body mass index (BMI): 27–35 kg/m^2^) were analysed. Eligible participants of the ACOORH trial were randomised with a 1:2 allocation ratio into either a lifestyle intervention control group (CON, *n* = 45) or a formula diet-based lifestyle intervention group (INT, *n* = 96). The study consisted of an initial intensity 26-week intervention phase, which was followed by a moderate intensity intervention phase until week 52. Participants were recruited in centers with long-term expertise in lifestyle and nutritional counselling, exercise intervention, as well as obesity therapy in Berlin, Dusseldorf, Essen, Frankfurt, Freiburg, Hamburg, Cologne, Munich (all Germany), Graz (Austria), London (Great Britain), and Strasbourg (France). The recruitment of the study started in January 2015 and the last patient out was in August 2017. The study was conducted in accordance with the ethical standards laid down in the 1964 Declaration of Helsinki and its later amendments. Approval of the research protocol was obtained from different ethics committees in each country of each participating center (primary responsible ethics committee is the Albert-Ludwigs-University of Freiburg, Germany (project identification code: 216/14)) and was registered at drks.de under the number DRKS00006811. All participants gave written informed consent prior to their inclusion in the study.

### 2.2. Study Population

Individuals without diabetes; aged 21–65 years; with a BMI of 27–35 kg/m^2^; a waist circumference (WC) of ≥88 cm (≥102 cm) for females (males); and, in addition, at least one of the following co-morbidities: (1) fasting blood glucose (FBG) 100–125 mg/dl, (2) triglycerides 150–400 mg/dl, (3) high-density lipoprotein (HDL)-cholesterol (HDL-C) < 40 mg/dl, or (4) untreated systolic (diastolic) blood pressure 140-160 (90–100) mmHg or anti-hypertensive medication were included in the multicenter ACOORH study. Participants were excluded when one of the following exclusion criteria was existent: (i) diabetes mellitus (FBG ≥ 126 mg/dl; HbA1c ≥ 6.5% (≥48 mmol/mol) or diabetes-related medical history (e.g., medical records or antidiabetic drugs)); (ii) total body weight > 141 kg; (iii) acute infections; (iv) chronic diseases such as cancer, chronic obstructive pulmonary disease, asthma, dementia, chronic gut diseases, psychoses, liver cirrhosis, nephropathy, and kidney insufficiency with glomerular filtration rate < 30 mL/min/1.73 m^2^; (v) plans to relocate to an area not served by the ACOORH; (vi) smoking cessation or planned smoking cessation during the study; (vii) drugs for active weight reduction; (viii) pregnancy or breast-feeding; and (ix) known intolerance with components of the used formula diet. In the present predefined subanalysis, only patients with prediabetes (HbA1c: 5.7–6.4% [39–46 mmol/mol]) were considered.

### 2.3. Intervention

Participants of both groups received quarterly lifestyle manuals containing information on healthy diet (limiting sweets, eating three times/day, being careful about the amount and composition of carbohydrates, eating whole-grain foods, fruits and vegetables, eating less fat, and limiting consumption of alcohol) as well as healthy behavior and were instructed to increase physical activity, but without further specifications regarding energy consumption. In order to improve compliance, participants in both groups were equipped with telemetric scales (smartLAB scale W; HMM Holding AG, Dossenheim, Germany) and pedometers (smartLAB walk P+; HMM Holding AG, Dossenheim, Germany) automatically transferring recorded data into a personalised online portal. These data could be monitored by both participants and study staff. At each subsequent visit, acquired data were discussed (i.e., steps and weight) and participants were further motivated to achieve their individual goals (i.e., weight goals, healthy lifestyle changes). Advice for increasing physical activity and strategies for self-motivation was also given.

Participants of the INT group additionally received a formula diet and an accompanying manual. The manual included information about the preparation of meal replacement as well as general facts about low-carbohydrate meals and their interaction with the blood glucose level. Participants of INT were instructed to document the amount of meal replacements consumed, the number of meals replaced, as well as the current weight and WC into their personal manual. At each visit, study nurses revised manuals and educated/instructed the participants of both groups in terms of a low-carbohydrate diet. Participants of the CON group received no further information, apart from the information already mentioned before, and were advised to measure their steps and weight daily.

**Figure 1:**
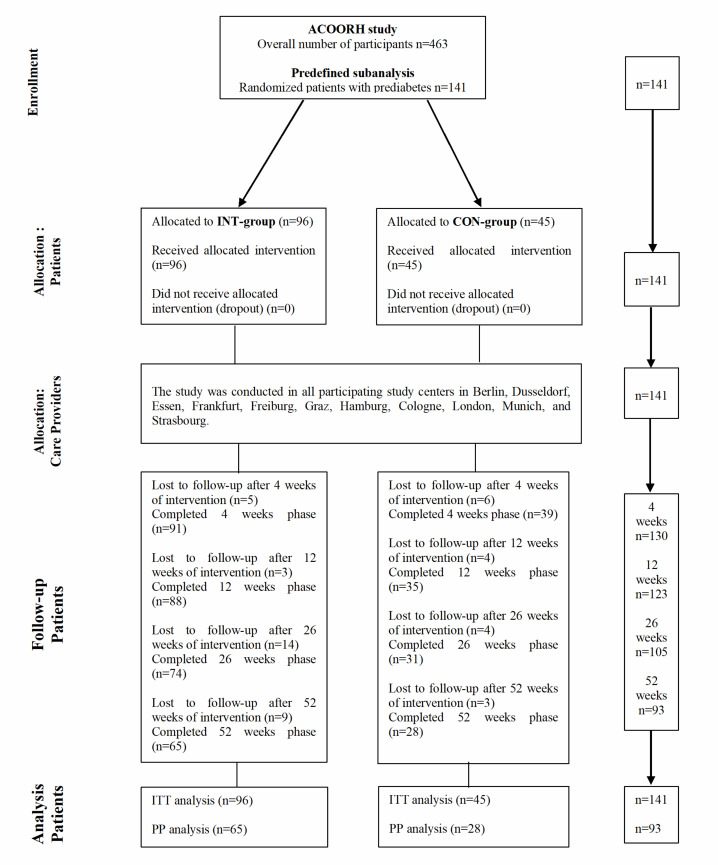
Flow diagram. ACOORH study, Almased Concept against Overweight and Obesity and Related Health Risk study; INT, intervention; CON, control; ITT, intention-to-treat; PP, per-protocol.

In addition, participants of both groups were asked to record a 4-day diet protocol (2× weekend days and 2× working days) at the 12- and 52-week follow-up. This information was used for lifestyle counseling. The quality of nutritional counselling was not evaluated. Study visits took place at baseline as well as after 4, 12, 26, and 52 weeks. A detailed timeline of the study visits is shown in Figure 1.

### 2.4. Diet Regimen

Meal replacement was performed as previously described [6]. In detail, a commercially available soy-yogurt-honey formula diet (Almased-Vitalkost^®^; Almased-Wellness-GmbH, Bienenbüttel, Germany) was provided to all participants of the INT group during the 26-week intervention phase and contained 30.6 g carbohydrates, 52.2 g protein, 1.8 g fat, and 1507 kJ (360 kcal) energy per 100 g powder. Participants were asked to replace breakfast, lunch, and dinner with 1 g powder/kg normal body weight (defined as height in cm—100) per meal dissolved in 250 mL water during the first week (~1200 kcal). Participants were further recommended to add 2–3 teaspoons (9–12 g) of safflower oil or rapeseed oil to the meal replacement. Energy-free beverages like water or unsweetened tea were allowed to be consumed ad libitum. No additional food was permitted. During weeks 2–4, participants replaced breakfast and dinner with the formula diet and ate a low-carbohydrate lunch (150–200 g of fish or meat, 500 g vegetables, and up to 50 g of carbohydrates from wholegrain bread or brown rice). The low-carbohydrate approach had to be continued in weeks 5–26 (1300–1500 kcal/day). Starting from week 5, participants of the INT group were instructed to preferably replace dinner with the formula diet. After 26-week follow-up, a cook book on low-carbohydrate meals and healthy cooking was provided to the INT group participants and they were advised to continue replacing one meal per day until the 52-week follow-up. We evaluated protocol compliance by requiring the participants to note the frequency and amount of formula diet they used during the first 26 weeks. This information in combination with the online data and the 4-day diet protocol were the basis of each counselling at the study visits.

### 2.5. Outcomes and Measurements

Anthropometrical, clinical (BMI, weight, WC, fat mass, lean body mass, systolic and diastolic blood pressure), and laboratory data were measured at baseline; after 4, 12, and 26 weeks of intervention; as well as after 52 weeks of follow-up. The assessors were blinded for group allocation. Body weight was measured in light clothing to the closest 0.1 kg, height to the closest 0.5 cm, and waist circumferences at the minimum abdominal girth (midway between the rib cage and the iliac crest). Body composition was examined using a body-fat scale (Seca medical Body Composition Analyzer^®^ (seca-mBCA), Hamburg, Germany). Blood pressure was measured after a five-minute rest in a sitting position on both arms. These measurements were performed manually as well as using validated equipment for central and peripheral blood pressure determination (Mobil-O-Graph PWA; I.E.M. GmbH, Stolberg, Germany). Venous blood was collected after an overnight fast and abdication of medication of at least 10 h, and laboratory parameters (HbA1c, FBG, total cholesterol, HDL and low-density lipoprotein (LDL)-cholesterol (LDL-C)) were analysed at local laboratories of each study center. Fasting insulin from all centers was measured according to general standards at the laboratory of SYNLAB in Stuttgart, Germany. Adverse and serious adverse events [23] were documented continuously.

### 2.6. Statistics

The sample size calculation of the ACOORH study was based on the ‘double-sided two sample analysis with continuity correction’ (SISA, simple interactive statistical analysis) method. The assumptions made for this calculation were based on a previous study [24], showing an average weight reduction of 9.0 ± 1.5 kg by consuming Almased^®^, while the control group achieved a loss of 6.0 ± 1.5 kg. The randomisation procedure was based on a block randomisation with block length six and allocation ratio 2:1. In order to measure such a weight reduction with a 2:1 randomisation, an accompanied power of 90%, and level of significance of 5%, 15 persons per group had to be recruited (CON, *n* = 5; INT, *n* = 10). Furthermore, an overall dropout rate of 20% was assumed and led to a sample size of at least 19 participants per group for each study center (*n* = 11).

Data are presented as means and standard deviations (mean ± SD), means and 95% confidence intervals (mean [95% CI]), or percentages, as appropriate. Per-protocol (PP) and intention-to-treat (ITT) analyses were performed. However, if not otherwise stated, the statistical calculation of each result was primarily based on the ITT approach. Missing values were imputed by the ‘last-observation-carried-forward’ (LOCF) principle.

In the present subanalysis, which focuses on the prediabetes cohort of the ACCORH study, the primary outcome is the prediabetes conversion rate to normoglycemia (defined as HbA1c <5.7% [<39 mmol/mol]) between both groups after 52 weeks. Secondary outcomes are on differences in weight change following the intervention after 12 and 52 weeks. Tertiary outcomes focused on within-group changes from baseline to week 12 and 52 regarding BMI, fat mass, lean body mass, WC, FBG, fasting blood insulin, HbA1c, triglycerides, systolic and diastolic blood pressure, total cholesterol, and HDL-C and LDL-C. These parameters were analysed using mixed models adjusting for repeated measurements, baseline values, and multiple testing. Non-parametric data were analysed with Mann–Whitney U, Wilcoxon, and Friedman test, and parametric data with Student’s *t*-test, paired *t*-test, and analysis of variance with repeated measures to determine differences between groups following the intervention. Multivariable univariate regression analyses were carried out to investigate group differences while adjusting for baseline parameters. Dichotomous variables as well frequencies were compared by Fishers exact test, McNemar test, or Cochrane Q test. All statistical tests were two-sided, and the level of significance was set at α = 0.05. *p*-values were adjusted for multiple comparisons using Bonferroni correction. All analyses were performed using SPSS 22.0 (SPSS Inc., Chicago, IL, USA) and GraphPad Prism 6.04 (GraphPad Software, San Diego, CA, USA). The statistical analysis was performed by an independent institute not involved in the study conductance (ACOMED statistik^®^, Leipzig, Germany).

## 3. Results

A total of 141 patients with prediabetes were analysed in this stratified analysis comprising *n* = 96 for the INT group and *n* = 45 for the CON group. One-hundred and twenty-three participants (87%, INT: *n* = 88 (88/96, 92%); CON: *n* = 35 (35/45, 78%)) and *n* = 105 (74%, INT: *n* = 74 (74/96, 77%); CON: *n* = 31 (31/45, 69%)) finished the 12- and 26-week intervention phase. Follow-up data after 52 weeks were available from 93 participants (66%, INT: *n* = 65 (65/96, 68%); CON: *n* = 28 (28/45, 62%)). Anthropometrical and clinical characteristics of both groups are shown in Table 1. Both groups were not statistically different in any parameter at baseline.

Furthermore, dropouts also showed no statistical difference in any characteristic compared with the non-dropout group (Supplementary Materials Table S1). The reasons for dropouts were as follows: (i) health problems, (ii) work-related reasons, (iii) personal reasons, and (iv) other reasons. No acute cardiac event, hospitalisation for cardiovascular disease, or other serious adverse event was noted.

**Figure 2:**
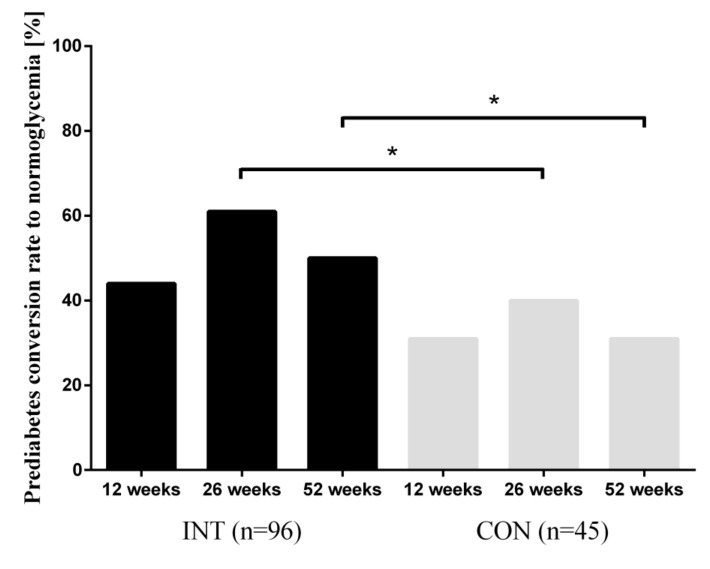
Prediabetes conversion rate to normoglycemia. HbA1c was determined at each follow-up and prediabetes was defined as HbA1c 5.7% to 6.5%. Data are presented as percentages. Analysis of differences in frequency distribution of prediabetes conversion to normoglycemia was calculated using Fisher’s exact test; * *p* < 0.05.

Compared with CON, significantly more participants of INT converted to normoglycemia after 52 weeks (50% vs. 31%; *p* < 0.05) in the ITT analysis, as shown in Figure 2. Furthermore, a significant difference could already have been shown at the 26-week follow-up (60% vs. 40%; *p* < 0.05) in the ITT analysis. As a consequence, the risk reduction led to a number-needed-to-treat of 5.3 for the INT-group after 52 weeks of intervention. The reduction in HbA1c was independent of weight loss.

Compared with CON, INT group reduced weight more significantly (−5.9 kg with 95% CI [−6.9; −4.9] vs. −2.4 kg [−4.0; −0.9]; *p* < 0.001) after 12 weeks in the ITT analysis. However, treatment superiority of INT over CON was not statistically significant after Bonferroni correction in the 52-week follow-up. The significant difference in weight reduction in the INT group was accompanied by improvements in BMI, FM, WC, FFM, LDL-C (all *p* < 0.01), HbA1c, and total cholesterol (both *p* < 0.05) after 12 weeks of intervention compared with CON in the between-group analysis. However, after Bonferroni correction (*n* = 30 comparisons, *p* < 0.0017), only BMI and FM remained statistically significant (both *p* < 0.0001). After the 52-week follow-up, only group differences for BMI, FM, and HbA1c remained statistically significant (all *p* < 0.05). These effects were lost after Bonferroni correction in the ITT analysis.

Changes of anthropometric and clinical parameters within both intervention groups after 12 and 52 weeks of intervention are also shown in Table 2. INT group demonstrated improvements in BMI, WC, FM, FBG, HbA1c, and systolic and diastolic blood pressure, as well as total cholesterol, HDL-C, LDL-C, and triglycerides after 12 weeks of intervention (all *p* < 0.01) in the ITT analysis. CON group improved in BMI, WC, FM, and HbA1c (all *p* < 0.05) in the ITT analysis. All changes were adjusted for multiple testing. From all these aforementioned improvements, INT group showed remaining long-term effects in BMI, WC, FM, HbA1c, total cholesterol, and LDL-C (all *p* < 0.01) after 52 weeks in the ITT analysis. CON group demonstrated remaining effects in BMI, WC, and HbA1c in the ITT analysis.

## 4. Discussion

The findings of the present study demonstrate that a low-intensity lifestyle intervention in combination with low-carbohydrate and energy deficit meal replacement therapy is superior to lifestyle intervention alone in improving prediabetes conversion rate to normoglycemia. Importantly, this finding remained significantly superior even after 52 weeks and was independent of weight reduction.

Our findings are in line with another uncontrolled lifestyle intervention program, which has also shown a reconversion rate of 40% from prediabetes (HbA1c: 5.7–6.49%) to normoglycemia (HbA1c: <5.7%) [21]. The present treatment regimen with a low-carbohydrate meal replacement is comparable to the recommendations for diets in type 2 diabetes, as well as recently published reviews and meta-analyses [25,26]. This approach is also supported by the current guidelines of the American Diabetes Association (ADA) [11] and the consensus reports of both ADA and the European Association for the Study of Diabetes (EASD) [12,13]. Furthermore, several studies have shown that a reduced carbohydrate intake [14] or a reduced number of carbohydrate-containing meals leads to improvements in glucose metabolism [15]. Findings of the landmark study, the PREDIMED trial [27], in which two high-fat/lower-carbohydrate Mediterranean diets were compared to a fat-reduced diet regarding the incidence of type 2 diabetes [28] as well as changes in anthropometry [29], support our carbohydrate-reduced formula diet approach.

The high prediabetes conversion rate to normoglycemia was accompanied by strong reductions in body weight and in fat mass. A recently published meta-analysis also showed comparable weight losses following very low-energy (<800 kcal per day) or low-energy liquid-formula (>800 kcal per day) diets (ranging from 8.9 to 15.0 kg) in people with obesity (BMI: 35.5–42.6 kg/m^2^) and without type 2 diabetes [30]. The difference in weight reduction compared with our trial can be explained by a higher calorie consumption per day (≈ 1300–1500 kcal per day) compared with the studies in the meta-analysis. We have chosen a moderate daily calorie target to reduce dropout rates and increase the participants’ therapy adherence. A recently published review indicates that a moderate weight loss is more sufficient for the transition from metabolically unhealthy obesity to metabolically healthy obesity with a lower risk for adverse outcomes in the long run rather than a rapid, severe weight loss [31], supporting our approach.

Further improvements were primarily achieved in the INT group in cardiometabolic parameters such as fat mass, HbA1c, fasting blood glucose, as well as systolic and diastolic blood pressure after 12 weeks of intervention. These results are comparable to other studies with low-energy diets in patients with type 2 diabetes [32,33] and in agreement with previous findings applying this formula diet in patients with prediabetes [5].

The strengths of the present study comprise (i) a relatively large number of patients studied per group who had prediabetes, (ii) a relatively long study period over 52 weeks, as well as (iii) the randomised controlled international and multicenter trial design with two intervention groups. Furthermore, we performed this study in a (iv) real-world setting with low-intensity intervention care in combination with a formula diet, as we had intended to design a practical intervention program, which could easily be implemented in present health care programs.

There are also limitations in our study that have to be appreciated. Firstly, we did not constantly use food diaries to control for decreased calorie consumption or false food compositions (e.g., amount of carbohydrates during diet). Owing to the well-known systematic errors associated with dietary records in an obese population, we had chosen not to constantly collect diet records during the whole study [34]. Furthermore, calorie targets or basal metabolic rates were not individually calculated to personalise treatment even further. However, participants of both groups were asked to conduct a 4-day dietary protocol in preparation for each study visit in order to improve lifestyle counseling. Furthermore, all participants were asked to note the number and amount of consumed formula diets and, therefore, we could at least assess consumption of formula diet within the first 12 weeks. Secondly, as this current subanalysis included only a part of the total ACOORH population, namely those patients with prediabetes, this stratified approach could have led to a selection bias. However, the analysis of the prediabetes data was predefined and belonged to the initial intention of the ACOORH study. Thirdly, another limitation of this subanalysis is the imputation of missing values by the LOCF method. This procedure is a conservative statistical approach to estimate treatment effects, which might have even underestimated our results.

Fourthly, we did not perform 2-h oral glucose tolerance test (OGTT) measurements to identify eligible participants to avoid inclusion of patients with diabetes. Inclusion in the study was only based on laboratory parameters accompanied with a confirming medical history record.

Our findings may not be generalisable, as the study size comprised *n* = 141 participants with an accompanied dropout rate of 34% after 52 weeks. However, we primarily applied the ITT analysis for our results, which takes the number of dropouts into account in its assumptions. We chose this more conservative approach to prevent overestimating our findings. To confirm our results, future studies need to be performed with larger sample sizes under comparable conditions. Furthermore, as the present study is a predefined subanalysis of the ACOORH trial, the power calculation was not based on an estimated conversion rate difference, but on an estimated weight loss difference as the primary topic of the main manuscript is weight management.

## 5. Conclusion

Overall, a low-intensity lifestyle intervention in combination with a low-carbohydrate and energy deficit formula diet leads to long-term prediabetes conversion rates to normoglycemia. Accompanied improvements comprise reductions in body weight, fat mass, waist circumference, fasting blood glucose, and other cardiometabolic risk factors, especially after the initial 12-week intensive intervention phase. These results support the therapeutic concept of low-carbohydrate and energy deficit diets with implemented formula meal replacement in patients with prediabetes when added to a lifestyle intervention program. Moreover, this therapy approach could be an effective treatment option for the prevention of type 2 diabetes and can be easily implemented in clinical practice and, therefore, efficiently scaled to a broader population.

## Figures and Tables

**Table 1:** Baseline characteristics. INT, intervention; CON, control.

	INT Group (*n* = 96)	CON Group (*n* = 45)	P
Sex (% male)	27.1	31.1	0.690
Age (years)	53 ± 9	52 ± 8	0.619
Weight (kg)	92 ± 14	92 ± 10	0.923
BMI (kg/m^2^)	32.2 ± 2.2	32.2 ± 2.3	0.900
WC (cm)	107 ± 9	108 ± 8	0.438
WHR	0.95 ± 0.08	0.96 ± 0.08	0.343
FM (kg)	38.1 ± 6.5	38.8 ± 6.4	0.566
FFM (kg)	53.7 ± 12.2	52.8 ± 8.8	0.665
HbA1c (%) (mmol/mol)	5.90 ± 0.22 41.0 ± 2.4	5.89 ± 0.21 41.0 ± 2.3	0.968
FBG (mg/dl)	101 ± 15	102 ± 11	0.560
FBI (uU/mL)	17.4 ± 10.4	15.9 ± 8.7	0.441
HOMA-Index	4.4 ± 2.7	4.1 ± 2.4	0.627
SBP (mmHg)	133 ± 12	133 ± 12	0.737
DBP (mmHg)	88 ± 11	89 ± 9	0.375
Total cholesterol (mg/dl)	221 ± 40	222 ± 41	0.948
HDL-C (mg/dl)	55 ± 13	54 ± 15	0.957
LDL-C (mg/dl)	142 ± 37	140 ± 40	0.838
Triglycerides (mg/dl)	148 ± 81	160 ± 77	0.406

Shown are means ± standard deviations or percentages. BMI, body mass index; DBP, diastolic blood pressure; FBI, fasting blood insulin; FBG, fasting blood glucose; FM, fat mass; FFM, fat free mass; HDL-C, high-density lipoprotein cholesterol; HOMA, homeostatic model assessment; LDL-C, low-density lipoprotein cholesterol; SBP, systolic blood pressure; WC, waist circumference; WHR, waist-to-hip ratio.

**Table 2:** Intra- and intergroup changes in the INT and CON groups after 12 and 52 weeks compared with baseline.

	12 Weeks			52 Weeks		
	INT	CON	P (INT vs. CON)	INT	CON	P (INT vs. CON)
Weight (kg)	−5.6 [−6.6; −4.5] ***	−1.9 [−3.5; −0.4] **	**<0.001**	−4.1 [−5.4; −2.8] ***	−2.3 [−4.2; −0.3] *	0.040
BMI (kg/m^2^)	−1.9 [−2.3; −1.6] ***	−0.7 [−1.2; −0.1] **	**<0.001**	−1.4 [−1.9; −1.0] ***	−0.8 [−1.5; −0.1] *	0.046
WC (cm)	−4.8 [−6.1; −3.4] ***	−2.3 [−4.3; −0.3] *	0.003	−4.0 [−5.7; −2.3] ***	−2.8 [−5.4; −0.3] *	0.223
FM (kg)	−4.5 [−5.4; −3.7] ***	−1.6 [−2.8; −0.3] **	**<0.001**	−3.2 [−4.3; −2.1] ***	−1.6 [−3.2; 0.1]	0.019
FFM (kg)	−1.0 [−1.5; −0.6] ***	−0.2 [−0.9; 0.4]	0.010	−0.9 [−1.4; −0.5] ***	−0.5 [−1.2; 0.1]	0.243
HbA_1c_ (%) (mmol/mol)	−0.19 [−0.25; −0.13] *** −2.1 [−2.7; −1.4] ***	−0.11 [−0.20; −0.02] * −1.2 [−2.2; −0.2] *	0.048	−0.19 [−0.25; −0.13] *** −2.1 [−2.7; −1.4] ***	−0.09 [−0.17; −0.01] * −1.0 [−1.9; −0.1] *	0.008
FBG (mg/dL)	−4.9 [−7.9; −1.9] ***	−2.0 [−6.4; 2.4]	0.068	−2.1 [−5.1; 0.8]	−3.8 [−8.1; 0.5]	0.471
FBI (uU/mL)	−2.4 [−5.5; 0.7]	−1.6 [−6.2; 3.0]	0.886	−1.7 [−5.0; 1.5]	−2.6 [−7.3; 2.2]	0.241
HOMA-Index	−0.75 [−1.60; 0.10]	−0.44 [−1.69; 0.80]	0.824	−0.51 [−1.30; 0.30]	−0.76 [−1.98; 0.45]	0.310
SBP (mmHg)	−6 [−10; −2] ***	−5 [−11; 1]	0.471	−3 [−6; 1]	−2 [−7; 3]	0.583
DBP (mmHg)	−3 [−5; −1] **	−4 [−7; −1] *	0.985	−2 [−4; 1]	−3 [−6; 1]	0.811
Total cholesterol (mg/dl)	−16 [−23; −9] ***	−6 [−17; 4]	0.027	−6 [−15; 2]	0 [−12; 12]	0.247
HDL-C (mg/dl)	−1 [−4; 1]	0 [−3; 3]	0.432	1 [−1; 4]	1 [−2; 4]	0.739
LDL-C (mg/dl)	−13 [−19; −7] ***	−3 [−12; 6]	0.007	−9 [−15; −2] **	−2 [−12; 8]	0.115
Triglycerides (mg/dl)	−15 [−33; 3] ***	−18 [−44; 8]	0.790	−7 [−27; 13]	−9 [−37; 20]	0.824

Data are shown as mean [95% confidence interval (CI)]. *** *p* < 0.001 vs. baseline; ** *p* < 0.01 vs. baseline; * *p* < 0.05 vs. baseline. All *p*-values were adjusted for multiple testing in the within-group analysis. Differences in changes after 12 and 52 weeks between both groups were analysed using mixed models adjusting for repeated measurements and baseline values (Bonferroni correction: *p* < 0.001677; bold written *p*-values represent significant difference). BMI, body mass index; DBP, diastolic blood pressure; FBI, fasting blood insulin; FBG, fasting blood glucose; FM, fat mass; FFM, fat free mass; HDL-C, high-density lipoprotein cholesterol; LDL-C, low-density lipoprotein cholesterol; SBP, systolic blood pressure; WC, waist circumference.

## Supplementary Materials

The following are available online at https://www.mdpi.com/2072-6643/12/7/2022/s1, Table S1: Baseline characteristics of the dropouts.
