# Supplementary material for: Prediabetes Conversion to Normoglycemia Is Superior Adding a Low-Carbohydrate and Energy Deficit Formula Diet to Lifestyle Intervention—A 12-Month Subanalysis of the ACOORH Trial
Source: Nutrients. 2020 Jul 7;12(7):2022. doi: 10.3390/nu12072022 (PMC7400892; doi:10.3390/nu12072022)
Supplement: Supplementary file 1 [file nutrients-12-02022-s001.pdf]

Supplementary Table 1. Baseline characteristics of the dropouts.

	INT-group (n=31)	CON-group (*n* = 17)	P
Sex (% male)	23.3	47.1	0.114
Age (years)	51±12	51±9	0.974
Weight (kg)	92±13	95±9	0.437
BMI (kg/m²)	32.0±2.3	33.1±1.7	0.095
WC (cm)	107±8	110±8	0.346
WHR	0.94±0.08	0.97±0.11	0.353
FM (kg)	38±7	39±6	0.543
FFM (kg)	53±10	55±9	0.552
HbA_{1c} (%)<br>(mmol/mol)	5.93±0.26<br>41.0±2.8	5.86±0.17<br>41.0±1.9	0.299
FBG (mg/dl)	101±15	104±11	0.543
FBI (uU/ml)	16.6±9.6	17.2±9.0	0.838
HOMA-Index	4.1±2.3	4.4±2.3	0.651
SBP (mmHg)	133±11	137±10	0.259
DBP (mmHg)	88±10	91±10	0.246
Total cholesterol (mg/dl)	214±41	215±46	0.978
HDL-C (mg/dl)	54±11	50±10	0.175
LDL-C (mg/dl)	136±38	142±44	0.643
Triglycerides (mg/dl)	141±58	140±58	0.953

Shown are means ± standard deviations, or percentages. BMI, body mass index; DBP, diastolic blood pressure; FBI, fasting blood insulin; FBG, fasting blood glucose; FM, fat mass; FFM, fat free mass; HDL-C, high-density lipoprotein cholesterol; LDL-C, low-density lipoprotein cholesterol; SBP, systolic blood pressure; WC, waist circumference; WHR, waist-to-hip ratio
